#  Rapid High Performance Liquid Chromatographic Method for Determination of Clarithromycin in Human Plasma Using Amperometric Detection: Application in Pharmacokinetic and Bioequivalence Studies 

**Published:** 2013

**Authors:** Seyed Mohsen Foroutan, Afshin Zarghi, Alireza Shafaati, Babak Madadian, Farshid Abolfathi

**Affiliations:** a *Department of Pharmaceutics, School of Pharmacy, Shahid Beheshti University of Medical Sciences, Tehran, Iran.*; b *Department of Pharmaceutical Chemistry, School of Pharmacy, Shahid Beheshti University of Medical Sciences, Tehran, Iran.*; c *Noor Research and Educational Institute, Tehran, Iran.*; d *Pharmaceutical Sciences Research Center, Shahid Beheshti University of Medical Sciences, Tehran, Iran. *

**Keywords:** Clarithromycin, Plasma, HPLC, Bioequivalency, Pharmacokinetic studies

## Abstract

A rapid, sensitive and reproducible HPLC method using amperometric detector was developed and validated for the analysis of clarithromycin in human plasma. The separation was achieved on a monolithic silica column (MZ- C8 125×4.0 mm) using acetonitrile-methanol-potassium dihydrogen phosphate buffer (40:6:54,v/v), with pH of 7.5, as the mobile phase at a flow rate of 1.5 mL/min. The assay enables the measurement of clarithromycin for therapeutic drug monitoring with a minimum quantification limit of 20 ng/mL. The method involves simple, protein precipitation procedure and analytical recovery was complete. The calibration curve was linear over the concentration range of 0.1-6 μg/mL. The coefficients of variation for inter-day and intra-day assay were found to be less than 6%. This method was used in bioequivalency and pharmacokinetic studies of the test (generic) product 2 × 500 mg clarithromycin tablets, with respect to the reference product.

## Introduction

Clarithromycin (Figure 1) is a semi-synthetic macrolide antibiotic derived from erythromycin with similar actions and uses. Because of its good antimicrobial activity against a wide range of Gram-positive and Gram-negative organisms, clarithromycin is used to treat the respiratory tract infections and skin and soft tissue diseases. Clarithromycin is rapidly absorbed from the gastrointestinal tract. Oral bioavailability of clarithromycin is about 55% and food has no effect on clarithromycin absorption (1, 2). Several analytical methods have been developed for determination of clarithromycin in human plasma. Some of those described HPLC methods utilizing positive ion electrospray ionization coupled to a tandem mass spectrometry (LC-MS/MS) detection (3- 6). These methods are very sensitive but they are not available for most laboratories because of their specialty requirement and financial reasons. In addition, some of the HPLC systems described methods utilizing ultra violet (UV) and electro chemical detectors (ECD) (7-12). Their Limits of quantification were not sensitive enough for pharmacokinetic studies and their sample preparations required derivatization or extraction steps and were time consuming.

The present study describes a rapid and validated HPLC method using a monolithic column with amperometric detection, which enables the determination of clarithromycin with good accuracy at low drug concentrations in plasma. Separation was performed on a reversed-phase column. The sample preparation only involves a simple one-step protein precipitation and no evaporation step is required. We also demonstrate the applicability of this method for bioequivalency and pharmacokinetic studies in humans.

## Experimental

*Chemicals*

Clarithromycin and Roxithromycin were supplied by Dorsa Daru Pharmaceutical Company (Dorsa Daru, Tehran, Iran). Clarithromycin is available as an oral tablet containing 500 mg of clarithromycin and other inactive ingredients. HPLC-grade acetonitrile and methanol and all other chemicals were obtained from Merck (Merck, Darmstadt, Germany). Water was obtained by double distillation and purified additionally with a Milli-Q system.

*Instruments and chromatographic conditions*

The chromatographic apparatus consisted of a model Wellchrom K-1001 pump, a model Rheodyne 7125 injector and Amperometric Detector +1.250 V, sens: 100 nA connected to a model Eurochrom 2000 integrator, all from Knauer (Berlin, Germany). The separation was performed on Chromolith Performance (MZ-C8 125 × 4.0 mm) column from Merck (Darmstadt, Germany). The mobile phase consisted acetonitrile- methanol-potassium dihydrogen phosphate buffer (40:6:54, v/v), with pH of 7.5, at a flow rate of 1.5 mL/min. The mobile phase was prepared daily and degassed by ultrasonication before use. The mobile phase was not allowed to recirculate during the analysis.

*Sample preparation*

To 500 μL of plasma in a glass-stoppered 15 mL centrifuge tube were added 50 μL of roxithromycin as internal standard (20 μg/mL) and 500 μL of acetonitrile. After mixing (30 s), the mixture was centrifuged for 10 min at 4000 rpm. Then 30 μL of supernatant was injected into liquid chromatography.

*Biological samples*

Twelve healthy male volunteers were included in this study. The study protocol was approved by the Ethics Committee of Shahid Beheshti University of Medical Sciences and written informed consent was obtained from the volunteers. Clarithromycin was administered in a double dose of 500 mg to the volunteers after overnight fasting. Plasma samples were collected at several intervals after dosing and then frozen immediately at - 20oC until assayed.

*Stability*

The stability of clarithromycin was assessed for spiked plasma samples stored at - 20°C for up to two months and at ambient temperature for at least 24 h. The stability of stock solutions stored at - 20°C was determined for up to one month by injecting appropriate dilutions of stocks in distilled water on day 1, 15 and 30 and comparing their peak areas with fresh stock prepared on the day of analysis. Samples were considered to be stable, if the assay values were within the acceptable limits of accuracy and precision.

*Plasma calibration curves and quantitation*

Blank plasma was prepared from heparinized whole-blood samples collected from healthy volunteers and stored at - 20°C. After thawing, 50 μL of clarithromycin working standards were added to yield final concentrations of 0.1, 1, 2, 3, 4, 5 and 6 μg/mL. 50 μL internal standard solution was added to each of these samples. The samples were then prepared for analysis as described above. Calibration curves were constructed by plotting peak area ratio (y) of clarithromycin to the internal standard versus clarithromycin concentrations (x). A linear regression was used for quantitation.

*Precision and accuracy*

The precision and accuracy of the method were examined by adding known amounts of clarithromycin to pool plasma (quality control samples). For intra-day precision and accuracy five replicate quality control samples at each concentration were assayed on the same day. The inter-day precision and accuracy were evaluated on three different days.

*Limit of Quantification (LOQ) and recovery*

For the concentration to be accepted as LOQ, the percent deviation from the nominal concentration (accuracy) and the relative standard deviation must be ± 10% and less than 10%, respectively, considering at least five-times the response compared to the blank response. The analytical recovery for plasma at three different concentrations of clarithromycin (1.4, 2.8 and 4.2 μg/mL) was determined. Known amounts of clarithromycin were added to drug-free plasma and the internal standard was then added. The relative recovery of clarithromycin was calculated by comparing the peak areas for extracted clarithromycin from spiked plasma and a standard solution of clarithromycin in water/methanol containing internal standard with the same initial concentration (six samples for each concentration level).

*Pharmacokinetic analysis*

Clarithromycin pharmacokinetic parameters were determined by noncompartmental methods. Elimination rate constant (K) were estimated by the least-square regression of plasma concentration-time data points lying in the terminal log-linear region of the curves. Half-life (T1/2) was calculated as 0.693 divided by K. The area under the plasma concentration-time curve from time zero to the last measurable concentration at time t (AUC0-t) was calculated using the trapezoidal rule. The area was extrapolated to infinity (AUC0-∞) by addition of Ct/K to AUC0-t where Ct is the last detectable drug concentration. Peak plasma concentration (Cmax) and time to peak concentration (Tmax) were derived from the individual subject concentration- time curves.

## Results and Discussion

Under the chromatographic conditions described, clarithromycin and the internal standard peaks were well resolved. Endogenous plasma components did not give any interfering peaks. Separation was performed on a reversed-phase column, which allows easy optimizing chromatographic conditions to obtain desirable resolution in a short time. Owing to use of the MZ-C8 column, much faster separations are possible as compared to traditional chromatographic columns packed. Accordingly, the chromatographic elution step is undertaken in a short time (less than 6 min) with high resolution. Figure 2 shows typical chromatograms of blank plasma in comparison to spiked samples analyzed for a pharmacokinetic study. The average retention times of clarithromycin and roxithromycin were 4.8 and 5.7 min, respectively. The peaks were of good shape and completely resolved from another at therapeutic concentrations of clarithromycin. In our method, sample preparation involves protein precipitation and no evaporation step is required. Protein precipitation became more efficient with increasing volumes of acetonitrile. However, greater volumes of acetonitrile diluted the sample, thereby affecting the sensitivity of the assay. To improve the sensitivity, a 1:1 ratio of acetonitrile to plasma was considered for sample preparation. Under this condition, the majority of protein was precipitated and clarithromycin and internal standard were free of inference from endogenous components in plasma. The calibration curve for the determination of clarithromycin in plasma was linear over the range 0.1-6 μg/mL. The linearity of this method was statistically confirmed. For each calibration curve, the intercept was not statistically different from zero. The correlation coefficients (r) for calibration curves were equal to or better than 0.999. The slops of plasma standard curves in the nine different preparations were practically the same (the CVs were less than 2% for the slopes of plasma standard curves). For each point of calibration standards, the concentrations were recalculated from the equation of the linear regression curves. The mean linear regression equation of calibration curve for the analyte was y = 0.669x + 0.0014, where y was the peak area ratio of the analyte to the internal standard and x was the concentration of the analyte. The analytical recovery for plasma at three different concentrations of clarithromycin was determined. Known amounts of clarithromycin were added to drug-free plasma in concentrations ranging from 1.4-4.2 μg/mL. The internal standard was added and the absolute recovery of clarithromycin was calculated by comparing the peak areas for extracted clarithromycin from spiked plasma and a standard solution of clarithromycin in methanol containing internal standard with the same initial concentration. The average recovery was 95.5 ± 0.58% (n = 6) and the dependence on concentration is negligible. The recovery of internal standard, roxithromycin was almost complete (98.9%) at the concentration used in the assay (20 μg/mL). Using amperometric detection method, the limit of quantification (LOQ), as previously defined, was 20 ng/mL for clarithromycin. This is sensitive enough for drug monitoring and other purposes such as pharmacokinetic studies. We assessed the precision of the method by repeated analysis of plasma specimens containing known concentrations of clarithromycin. As shown in Table 1, coefficients of variation were less than 2.2%, which is acceptable for the routine measurement of clarithromycin. Stability was determined for spiked plasma samples under the conditions as previously described. The results showed that the samples were stable during the mentioned conditions. The aim of our study was to develop a rapid and sensitive method for the routine analysis of biological samples in pharmacokinetic and bioequivalence studies of clarithromycin. This method is well suited for routine application in the clinical laboratory because of the speed of analysis and simple extraction procedure. Over 300 plasma samples were analyzed by this method without any significant loss of resolution. No change in the column efficiency and back pressure was also observed over the entire study time, thus proving its suitability. In this study plasma concentrations were determined in twelve healthy volunteers, who received 1000 mg of clarithromycin each. The mean values for the variable Cmax were 4.2 μg/mL. The mean values for the variable AUC were 39.6 h μg/mL. Figure 3 shows the mean plasma concentration-time profile of clarithromycin. The derived pharmacokinetic parameters of 12 healthy volunteers are summarized in Table 2. These pharmacokinetic parameters are in good agreement with that found previously (1, 2).

## Conclusion

A rapid and simple HPLC method has been described for the analysis of clarithromycin in plasma. Using MZ-C_8 _column, the chromatographic elution step is undertaken in a short time (< 6 min) with high resolution. In addition, the use of a simple one-spot sample preparation instead of more complex extraction procedures makes this method suitable for pharmacokinetic and bioequivalence studies of clarithromycin in humans.
